# The Latest Treatment of Phthisis

**Published:** 1887-03

**Authors:** 


### *THE LATEST TREATMENT OF PHTHISIS.*

The very latest treatment of phthisis is by large doses of tannin. Scarcely had we begun to grasp the details of the medicated gaseous enemata process, as practiced in the French hospitals, before Drs. Arthaud and Raymond announce something simpler and better. We learn from the Paris letter to the *British Medical Journal*, that these gentlemen began by experimenting on rabbits, in order to find some substance which would render them non-susceptible to inoculations of tuberculous matter. They obtained some very remarkable results from tannin.

"Six rabbits were treated for a month with doses of tannin, varying from seven and a-half to fifteen grains; after two successive inoculations, one with lung-tissue from a patient who had died of acute tuberculosis, the other with miliary tubercle from a hospital patient—no trace of infection was observed, while the three other rabbits, to which tannin had not been given, succumbed in consequence of inoculations with the same material. These experiments suggested a mode of treatment which has been adopted with excellent results in over fifty cases. Tannin was given in doses of from thirty to sixty grains a day, and the improvement was visible at the end of a fortnight, the patients had increased in weight, and no relapse occurred. In cases of acute tuberculosis, both in children and adults, it sometimes happens that the symptoms appear less favorable; but at the end of a week or a fortnight, the patient's condition improves, even when fatal results have been feared. From these experiments the following conclusions may be drawn: (1) The tannin is preferable to sulphide of carbon or iodoform in the treatment of tuberculosis; (2) that animals submitted to this treatment for a month offer great resistance to the action of tubercular virus."—*Southwestern Medical Gazette.*

